# Plasma and Whole Blood Taurine Concentrations in Dogs May Not Be Sensitive Indicators of Taurine Deficiency When Dietary Sulfur Amino Acid Content Is Reduced

**DOI:** 10.3389/fvets.2022.873460

**Published:** 2022-05-09

**Authors:** Cristina L. Tôrres, Vincent C. Biourge, Robert C. Backus

**Affiliations:** ^1^Department of Molecular Biosciences, University of California, Davis, Davis, CA, United States; ^2^Royal Canin SAS Research Center, Aimargues, France; ^3^Department of Veterinary Medicine and Surgery, University of Missouri, Columbia, MO, United States

**Keywords:** dilated cardiomyopathy (DCM), taurine, methionine, cysteine, bile acids, antibiotics, dog size

## Abstract

**Background:**

Taurine status is impacted by dietary supply of methionine and cysteine (SAA) and possibly intestinal microbial activity, where plasma and whole blood taurine concentrations are currently used to evaluate taurine status.

**Objective:**

We determined effects of dietary SAA restriction on rate and extent of taurine depletion of blood and skeletal muscle in dogs of two body sizes, and whether oral antibiotic administration affected the taurine depletion and fecal bile acid excretion of the dogs.

**Methods:**

Adult, male, Beagles (*n* = 6; 10.1–13.1 kg) and larger mixed-breed dogs (*n* = 6; 28.5–41.1 kg) were given four dry-expanded diets, whereby each successive diet contained lower protein and/or SAA concentration. After receiving the final diet for 44 weeks, all dogs were orally administered a mixture of ampicillin, neomycin sulfate, and metronidazole for 12 weeks. Taurine concentrations were determined every 2–4 weeks in venous blood and voided urine and every 4 to 16 weeks in biopsied semimembranosus muscle. Fecal bile acid excretion before and after antibiotics administration were quantified.

**Results:**

When given for 36 weeks the lowest SAA diet, 3.4% methionine and 2.9% cystine, taurine concentrations in whole blood were not different between groups, while taurine in plasma declined (*P* < 0.05) in large but not in small dogs, and taurine in biopsied muscle decreased (*P* < 0.05) by 50% in large and by 37% in small dogs. Concentrations of taurine in muscle were lower (*P* < 0.01) and fecal bile acids greater (*P* = 0.001) in large than small dogs. Antibiotic administration restored plasma and muscle taurine to initial concentrations and halved fecal bile acid excretion by dogs of both groups.

**Conclusions:**

Blood taurine concentration may not be a sensitive indictor of taurine depletion caused by low intake of bioavailable SAA in dogs, especially in large dogs. Taurine status and dietary SAA requirements of dogs may substantively depend on taurine loss mediated by intestinal microbiota.

## Introduction

Heart failure resulting from a dilated cardiomyopathy (DCM) and induced by feeding certain commercially available diets was first described in domestic cats in 1987 (1). The condition was uniquely associated with low plasma taurine concentrations and reversed in many cats by oral taurine supplementation and change of diet (2). At the time, research had already shown that taurine was an essential nutrient for cats (3). Inadequate synthesis of taurine from the dietary sulfur amino acids (SAA), methionine and cysteine, was known to cause an irreversible retinopathy in cats (4). Others had shown that dietary taurine was essential for normal growth, development, and reproduction of cats (5). Several hypotheses about dietary factors causing the DCM in cats were investigated during the following 15 years. Heat processing (6), sources and amount of dietary fiber (7), and protein source, quantity, and quality (8, 9) are among factors shown to significantly impact circulating taurine concentrations in cats. Differences in dietary matrix were observed to vary dietary taurine requirement of cats over a 6-fold range (10). Dietary taurine requirements were lowest with purified ingredients and greatest with heat-processed canned foods. Morris and others (11) postulated that dietary need for taurine was largely dependent on gastrointestinal microbial-mediated loss of taurine. Kim and colleagues (8) observed marked improvement in the taurine status of cats given antibiotics. They also found that antibiotic ingestion reduced fecal bile acid excretion and fecal cholyltaurine hydrolase activity. The bile acids of cats, as well as dogs, are conjugated in liver almost exclusively with taurine (12, 13). This observation supported postulation that dietary taurine requirement in cats is influenced by modulation of enterohepatic circulation of taurine in bile acids (14, 15). A kinetic study using a dual radiolabeled bile acid in cats indicated that dietary matrix substantially impacts bile reabsorption and showed only 21–34% of taurine in taurocholic acid being recycled (14). Using different methods, research in humans indicated a wide range in small intestinal bile acid reabsorption percentage, 49–86% (16). Thus, fractional reabsorption of taurine-conjugated bile acids may be affected substantively by intestinal microbial activity.

Investigators credited with identifying the diet-associated DCM in cats speculated that the condition might also occur in other species (1). However, they conceded it unlikely because cats seemed unique among domestic species in having a dietary need for taurine. In the following years, low plasma taurine concentrations were sporadically reported in privately-owned dogs that were diagnosed with DCM (17–21). This finding was surprising as affected dogs were maintained on commercial diets which were apparently nutritionally adequate. Physiological needs for taurine in dogs are met through synthesis from methionine and cysteine when these precursors are sufficiently abundant and bioavailable. Taurine deficiency and reduced cardiac function has been reported in dogs maintained on low SAA diets (22). Low bioavailability of SAA in diets given to some dogs with DCM was suggested to explain coinciding taurine deficiency (23, 24). Larger dogs appeared more affected than small dogs (19). Among large dogs diagnosed with taurine-deficiency DCM, there was no clear association with breed or genetic background. This finding contrasted with known breed risk for DCM at the time (25) and which continues to be observed (26).

Within the past 5 years, reports of diet-associated DCMs in dogs have increased (27, 28). A remarkable finding has been a frequent association of DCM with commercial diets containing ingredients not historically used by dog food manufacturers. Implicated diets reportedly contained large amounts of pulses, tubers, and/or proteins from exotic sources such as kangaroo, bison, and duck (29). Unlike in prior years, taurine deficiency in these dogs is not as clear. Most often, blood and plasma taurine concentrations are not low or low enough to clearly indicate taurine deficiency (28). Nevertheless, taurine supplementation has been given to these dogs along with dietary change and conventional medical management. These interventions have been effective unless cardiac decompensation on initial presentation was too severe (26, 27).

To our knowledge, kinetics of taurine depletion in dogs have not been described in the literature. When given a nutritionally complete and highly digestible diet, a surfeit of taurine production from dietary SAAs likely occurs in dogs, while in cats little taurine is synthesized. Declines in taurine concentrations of blood or plasma may not well reflect changes in myocardium or other tissues of dogs. When dietary taurine is lacking in cats, plasma taurine concentration decreases rapidly, by more than half in 3 weeks; whereas, in the skeletal muscle of cats where the largest body pool of taurine resides, taurine appears conserved and depletes at a much slower rate, declining by half only after 4 months (30). We question whether taurine depletion may not be as readily detected in dogs as it is in cats. A study, reported as an abstract, found in laboratory Beagles fed a very low protein diet for 24 months indicated that taurine concentration in sampled blood may not well reflect taurine status of skeletal and cardiac muscle (31).

In considering past and recent observations of diet-associated DCM in dogs, we postulated that interactions of diet matrix with gastrointestinal microflora may cause development of DCM in dogs, as postulated for cats, while the kinetics of taurine depletion may differ between dogs and cats. To investigate this possibility, the taurine status of dogs of two different body sizes was assessed through measurement of taurine in whole blood, plasma, biopsied skeletal muscle, and urine. The dogs' dietary SAA concentration was progressively reduced until circulating taurine concentrations declined, but without reaching levels that have been associated with taurine-deficiency DCM in dogs. We hypothesized that relative rates of taurine depletion in the sampled tissues would differ from those reported in cats fed taurine free diets. Thereafter, the effects of a broad-spectrum mixture of antibiotics on taurine status and fecal bile acid excretion were assessed. Our hypothesis was that the antibiotics would improve taurine status and reduce bile acid excretion as in cats.

## Materials and Methods

The research was conducted between January of 2001 and July of 2003 in two consecutively undertaken experiments: a study to determine whether a diet reduced in SAA and fed to maintain an ideal body condition affected the taurine status in dogs of two different body sizes (Experiment 1), and a study to determine whether addition of oral antibiotics impacted taurine status of the dogs, and the amount of bile acids they excrete when dietary SAA concentration is low (Experiment 2).

### Animals

Twelve university-owned adult (2–4 y old) male dogs (11 sexually intact, 1 neutered) were assigned to two groups according to body size. One group was designated small dogs (SD) and consisted of 6 Beagles of median (range) body weight of 11.6 (10.1–13.1) kg. The other group was designated as large dogs (LD) and consisted of 6 mixed-breed dogs of median (range) body weight of 35.5 (28.5–41.4) kg. All dogs were considered healthy based on physical examination and cardiac ultrasound findings, body condition assessment, results of fecal parasite and heartworm testing, as well as clinical hematological and serum biochemical observations. While studied, the dogs were socialized daily, except on the days of feces collection. The dogs were individually kenneled in large outdoor runs with concrete floors that allowed for visual social interaction and space sufficient for physical activity precluding need for scheduled exercise. The runs were partially roofed and fitted with overhead heating and side panels to provide shelter from direct sun, rain, wind, and temperature extremes. Water was available at all times. Diet was presented once daily in amounts deemed adequate for maintaining initial body weight and condition. Protocols for use of the dogs (8,840 and 9,550) were approved by the Animal Use and Care committee at the University of California, Davis and complied with the NRC guidelines for research animals.

### Diets

Initially all dogs received for 4 weeks a commercially available diet (Table 1, “Adapt”, Royal Canin, Size Medium Adult, Royal Canin, Aimargues, France). This diet met or exceeded the nutrient profiles for canine maintenance as recommended by the Association of American Feed Control Officials (AAFCO) (32). Thereafter, the dogs received in sequence four custom manufactured extruded dry-type diets (Table 1, Diets “1”, “2”, “3”, and “4”). The custom diets contained the same ingredients but with differing ingredient proportions. These diets were formulated to meet or exceed the dry matter nutrient profiles recommended by the AAFCO (32) for canine maintenance, except for concentrations of crude protein, which for the last two diets were less than the recommendation (15.7 and 15.6% vs. 18%) (Table 1). As protein concentrations in the diets decreased, a metabolizable energy (ME), caloric equivalence in dietary fat was added. The experimental diets were high in crude fiber as a result of inclusion of rice bran, which was maintained at approximately 27% of diet dry weight. Dietary ME on a kcal/g basis was estimated using modified Atwater coefficients in the equation: 10 × (crude protein % × 3.5) + (crude fat % × 8.5) + (nitrogen-free extract % × 3.5). Digestibility of the SAA of the diets was determined by a commercial laboratory using a cecectomized rooster assay (Guyomarc'h, Vannes, France). Amino acid digestibility indicated by the rooster assay reportedly correlates well with digestibility observed in ileal cannulated dogs (33).

**Table 1:** Diet period durations, dry matter compositions, and nutrient digestibilities^a, b^.

	**Diet**				
**Duration**	**Adapt**	**1**	**2**	**3**	**4**
Week	4	16	28	8	44
**Nutrient** ^**c**^					
Crude protein, g/kg	278	224	185	157	156
Crude fat, g/kg	134	121	136	218	232
Crude fiber, g/kg	23	28	31	29	19
Ash, g/kg	63	96	89	79	83
ME, Mcal/kg	3.87	3.67	3.69	4.16	4.30
Protein ME, %	25.1	21.4	17.5	13.2	12.7
SAA, g/kg	8.5	7.4	6.1	8.5	6.0
Methionine, g/kg	5.6	4.3	3.4	3.6	3.4
Cystine, g/kg	2.9	3.1	2.7	4.9	2.6
SAA apparent digestibility^d^, g/kg	nd	6.1	4.6	7.5	4.7
SAA true digestibility, %	nd	81.9	75.0	87.8	77.9
Methionine true digestibility, %	nd	87.8	84.0	88.3	85.0
Cystine true digestibility, %	nd	73.8	63.0	87.3	68.6
Taurine, g/kg	1.02	0.23	0.22	0.26	0.28

*Adapt, adaptation; ME, metabolizable energy; SAA, sulfur amino acid; nd, not determined*.

a *Ingredients of the commercial, adaptation diet in decreasing order of weight were rice, chicken and chicken by-product, corn, chicken fat, beet pulp, minerals and vitamins, fish meal, salmon oil, yeast, egg*.

b *Ingredients of the experimental diets in decreasing order of weight were brewers' rice, rice bran, lamb-meal, poultry fat, lamb digest, proprietary vitamin and mineral mixtures*.

c *Concentrations expressed on a dry matter basis*.

d *Digestibilities determined with cecectomized rooster assays*.

### Experiment 1

Prior to the start of Experiment 1, all dogs were maintained on the commercially available diet for 4 weeks. Thereafter, the experimental diets, Diet 1, Diet 2, Diet 3, and Diet 4, were sequentially presented for periods of 16, 28, 8, and 44 weeks, respectively. Experiment 1 lasted 96 weeks in total. Although the experimental diets were not supplemented with taurine, each contained a low background amount of taurine (Table 1). With successive introductions of the experimental diets, further reduction of dietary SAA was intended. However, this was not achieved with Diet 3, which was introduced before results of SAA analysis were received. The amount of diet presented daily to each dog was sufficient to maintain their body condition score (BCS) near ideal, where a score of 5 on a 9-point scale was considered ideal (34). Recording of food intake began after week 16 while Diet 1 was presented and continued monthly thereafter until end of the experiment. Daily ME intakes (kcal/day) were calculated by multiplying daily food intake (g/day) by diet ME density (kcal/g diet). Body weights and BCSs of the dogs were determined once to two times per month and dietary allowances were adjusted as needed for BCS maintenance. Heparinized, cephalic venous blood (3 mL) was collected by venipuncture after overnight (~16 h) food withholding, every 2–4 weeks, for determination of taurine concentrations in whole blood and extracted plasma. Urine was collected during voluntary voiding during weeks 0, 2, 16, 28, 44, 52, 60, 64, 68, 96. Semimembranosus muscle of the dogs was biopsied during weeks 0, 4, 16, 20, 28, 36, 52, 60, 68, 80, and 92, throughout the feeding of the four experimental diets. For biopsies, dogs were sedated, and then anesthetized with isoflurane, and ~20–40 mg of the muscle was aseptically obtained with a 12-ga percutaneous biopsy needle. Post-procedure wound inspection, as well as clinical signs and behavior, were assessed by a veterinarian, and treated as needed. Collected samples of blood, plasma, urine, and muscle were stored at −20°C pending analyses.

### Experiment 2

Following Experiment 1, all dogs continued to receive Diet 4 for an additional 12 weeks in amounts sufficient to maintain their body weights. During this time, each dog received twice daily, per os, a mixture of antibiotics commonly used in treatment of antibiotic responsive enteropathies. The dosages used were 22 mg/kg of ampicillin (Principen^®^ capsules, Apothecon, Princeton, NJ, USA), 20 mg/kg of neomycin sulfated (Biosol^®^ liquid, Pharmacia & Upjohn Company, Kalamazoo, MI, USA), and 15 mg/kg of metronidazole (Metronidazole tablets, Teva Pharmaceuticals, Sellersville, PA, USA). The dosages were within the ranges reported as effective for treatment of enteric or systemic infections of dogs (35).

During Experiment 2, venous blood was collected at weeks 0, 4, 8, and 12 as previously described. Whole blood and plasma aliquots of the samples were stored frozen (−20°C) for later analysis. Semimembranosus muscle was biopsied at weeks 0 and 12 of Experiment 2. The biopsies were obtained and retained as described in Experiment 1. Before the beginning and during the last week of antibiotic treatment, the total of feces passed from each dog on run floors were collected daily for 5 consecutive days and stored at −20°C. Feces from each dog were pooled by collection period and stored at −20°C for later dry matter and bile acid content determinations.

### Laboratory Analyses

Plasma and whole blood taurine concentrations were determined as previously described (30). For this, plasma was extracted in an equal volume of 60 g/L of sulfosalicylic acid solution and centrifuged at 3,900 x g for 10 min. Whole blood was frozen and thawed twice and diluted 1:1 in distilled water. The diluted mixture was extracted as described for plasma. Urine was extracted as described for plasma. For stability of taurine in storage, supernatants of the sulfosalicylic acid extracts of urine, plasma, and blood were frozen at −20°C until taurine concentrations were later determined with an automated amino acid analyzer (Model 121-MB, Beckman Instruments, Fullerton, CA, USA).

Biopsied muscle samples were mixed with distilled water and sonicated to disrupt cells. The muscle homogenate was extracted in sulfosalicylic acid solution, fractionated by centrifugation, and taurine concentration in resulting supernatant determined after thawing, as described for plasma.

After the experiment, total bile acid concentration in fecal dry matter was determined by previously described methods (36). For this, frozen feces collected from each dog were thawed, pooled by 5-day collection period, homogenized in water, weighed, and a portion dried for estimation of daily dry matter excretion. For bile acid analysis, dried homogenate residue (0.1 g) was heated for 120 min in 1.0 mL of potassium hydroxide-ethylene glycol solution (40 g/L). After cooling, and dilution with 1.0 mL of aqueous sodium chloride solution (20 g/L), and acidification with concentrated (370 g/L) hydrochloric acid (0.2 mL), bile acids in the resulting mixture were extracted into 6 mL of diethyl ether. Following evaporation of the ether, the extracted bile acids were dissolved in 3 mL of methanol and quantified with a commercial kit that measured bile acid mediated reduction of NAD in the presences of 3α-hydroxysteroid dehydrogenase (No. 450, Sigma Chemical, St Louis, MO, USA).

### Statistical Analyses

The significance of effects of dog size and dietary condition were assessed on the variable observations—body weight, ME intake, SAA intake, fecal bile acid concentration and excretion, ratio of taurine to creatinine in urine, and concentrations of taurine in plasma, whole blood, and semimembranosus muscle. Significance was determined with a repeated-measures, mixed-model ANOVA where fixed effects were dog size (SD and LD), dietary condition (adaptation, Diet 1, Diet 2, Diet 3, Diet 4, and Diet 4 with oral antibiotics), and interaction between dog size and diet while random effects were the dog subjects. Tukey-adjusted, multiple comparisons were used when significance was found for dietary condition. Variable observations were considered normal when means and medians of the observations were within 10% of each other and skew and excess kurtosis of the observations each were between −1 and 1. Because daily ME intakes depended on body size and therefore were bimodally and not normally distributed, the observations were normalized by transformation to percentages of values recorded while the first experimental diet (Diet 1) was presented. Logarithmic transformation was required to standardize observations on intakes of dry matter, ME, and SAA expressed on a metabolic body weight basis.

Effects of oral antibiotic on taurine concentrations in plasma, whole blood, urine, and semimembranosus muscle were assessed from significance of differences in observations just prior to and during antibiotic administration. For this, a repeated-measures, mixed-model ANOVA was used to determine significance of differences with sampling time, where dog size, week of sampling, and interaction of dog size and sampling week were fixed effects and subject dogs were random effects. Logarithmic transformation was required to standardize observations on daily fecal bile acid excretion. When significance was found, differences between weeks were identified using Tukey-Kramer adjusted, *post-hoc* multiple comparisons.

For each dog group, the significance of difference in body weight before and after antibiotics administration was determined by paired *t*-test analysis. The significance of differences between initial and final body condition scores was determined with sign tests.

The significance of correlations between normalized daily SAA intake and taurine concentrations in plasma, whole blood, and semimembranosus muscle were determined with Spearmen-rank order correlation analyses.

Statistical software was used to conduct the analyses (SAS 9.4, SAS Institute, Cary, NC, USA). Outcome variable observations are reported as means and their variances expressed as SEM. Effects were considered significant if *P* < 0.05.

## Results

### Experiment 1

The BCSs of all dogs ranged from 5 to 7 out of 9. The BCS of LD and SD were similar during the experiment and were not significantly different between beginning and end of the experiment. Body weights of LD were approximately 3-times greater than those of SD (Table 2). Across the experimental diet periods, body weights differed. When Diets 3 and 4 were presented, SD body weights were greater (*P* ≤ 0.04) by 8 and 15%, respectively, than when Diet 1 was received. Body weights of the LD when Diet 2 was given was slightly greater (*P* < 0.03) by 4–7% than when the adaptation and Diets 1 and 4 were presented, and not different from when Diet 3 was given.

**Table 2:** Body size and diet effects on body weight, daily intakes of metabolizable energy and sulfur amino acids, and taurine concentrations in plasma, whole blood, semimembranosus muscle, and urine of 6 small dogs and 6 large dogs.

	**Size**	**Diet**						***P*-value**		
**Variable^**1**^**		**All**	**Adapt**	**1**	**2**	**3**	**4**	***S***	***D***	***S × D***
Body weight, kg	Small	12.9 ± 0.1	12.1 ± 0.3^a^	12.1 ± 0.3^a^	12.3 ± 0.3^a^	13.0 ± 0.4^a, b^	13.9 ± 0.3^b^	<0.01	<0.01	<0.01
	Large	36.5 ± 0.3^*^	35.8 ± 1.6^a^^*^	35.3 ± 1.6^a^^*^	37.7 ± 1.5^b^^*^	36.1 ± 1.6^a^^*^	36.2 ± 1.5^a^^*^			
**Daily intake**										
ME, Mcal	Small	1.26 ± 0.07	nd	1.21 ± 0.11^a, b^	1.38 ± 0.75^a^	1.35 ± 0.09^a^	1.11 ± 0.07^b^	<0.01	<0.01	<0.01
	Large	2.18 ± 0.07^*^	nd	2.60 ± 0.11^a^^*^	2.30 ± 0.07^b^^*^	1.83 ± 0.09^c^^*^	1.97 ± 0.07^c^^*^			
ME, %^2^	Small	101 ± 1	nd	100 ± 2^a, b^	103 ± 1^a^	102 ± 1^a^	98 ± 1^b^	<0.01	<0.01	<0.01
	Large	96 ± 1^*^	nd	100 ± 2^a^	97 ± 1^a^^*^	92 ± 1^b^^*^	94 ± 1^b^^*^			
ME, kcal/kg^0.75^	Small	186 ± 7	nd	185 ± 11^a, b^	209 ± 7^a^	196 ± 9^a^	156 ± 7^b^	<0.01	<0.01	<0.01
	Large	148 ± 7^*^	nd	180 ± 11^a^	152 ± 7^a^^*^	128 ± 9^b^^*^	134 ± 7^b^			
SAA, mg/kg^0.75^	Small	254 ± 9	nd	228 ± 17^*a*^	274 ± 9^*a*^	344 ± 13^b^	170 ± 8^c^	<0.01	<0.01	<0.01
	Large	197 ± 9^*^	nd	221 ± 17^a^	196 ± 9^a^^*^	225 ± 13^a^^*^	146 ± 8^b^			
**Taurine**										
Plasma, μmol/L	Small	89 ± 5	94 ± 6	93 ± 6	86 ± 5	80 ± 7	90 ± 5	0.08	<0.01	<0.01
	Large	76 ± 5	93 ± 6^a^	79 ± 6^a, b^	75 ± 5^b^	75 ± 7^a, b, c^	62 ± 5^c^^*^			
Whole blood, μmol/L	Small	301 ± 13	309 ± 17	326 ± 15	297 ± 14	275 ± 16	300 ± 13	0.13	<0.01	<0.01
	Large	273 ± 13	322 ± 16^a^	275 ± 15^b^	282 ± 14^b^	244 ± 16^c^	240 ± 13^c^			
Muscle, μmol/g	Small	15.4 ± 0.7	17.7 ± 1.3^a, b^	13.2 ± 1.3^a, c^	14.5 ± 1.3^a^	20.2 ± 1.8^b^	11.5 ± 1.0^c^	<0.01	<0.01	0.17
	Large	9.5 ± 0.7^*^	10.8 ± 1.3^*^	9.5 ± 1.3	9.8 ± 1.3	10.8 ± 1.8^*^	7.2 ± 1.0			
Urine^3^, mmol/mmol	Small	0.50 ± 0.07	0.70 ± 0.11	0.25 ± 0.14	0.42 ± 0.10	0.65 ± 0.15	0.48 ± 0.08	0.02	0.05	0.67
	Large	0.18 ± 0.08^*^	0.27 ± 0.15	0.15 ± 0.15	0.11 ± 0.11	0.28 ± 0.15	0.11 ± 0.09			

*S, size; SE, standard error; D, diet; ME, metabolizable energy; nd, not determined*.

1 *Values are least squares means ± standard errors*.

2 *Percentage of ME intake (Mcal/day) relative to ME (Mcal/day) intake during week 20, when ME intakes were first recorded and Diet 1 had been given for 16 weeks*.

3 *mmol of taurine per mmol of creatinine*.

* *Significantly different from small dog value. Letter annotated values that do not share a common letter differ (P < 0.05) from other values with in the row*.

Because daily ME intakes of LD were approximately twice those of SD (Table 2), the ME intakes were evaluated after normalization by dog metabolic weight, i.e., body weight in kilograms to the 0.75 power. Normalized ME intakes of LD were decreased (*P* < 0.01) by a mean of approximately 25% when on Diet 4 compared to Diet 1. The decrease may have been related to seasonal temperature differences, as energy intake of dogs is increased in cool conditions (37). Although the dogs were provided heated and covered shelter, they had voluntary access to outdoor runs. Diet 1 was introduced during January while Diet 4 was introduced during April, when reported mean (range) temperatures are 7 (3–12) °C and 16 (8–23) °C, respectively. Normalized ME intakes of LD were less (*P* < 0.01) than those of SD when Diets 2 and 3 were presented, however, significant differences between the dog groups were not found when Diets 1 and 4 were consumed (Table 2).

Normalized daily intakes of SAA (mg/kg^0.75^/day) were calculated by multiplying daily food intake (g/day) by corresponding diet SAA concentration (mg/g) and dividing by metabolic body weight (kg^0.75^). Across diets, normalized SAA intakes differed with more variation occurring in the SD compared to LD (Table 2). However, when the first and last experimental diets (Diets 1 and 4) were presented, the normalized SAA intakes were not significantly different between the dog groups. The mean of normalized daily SAA intakes when Diet 4 was given was approximately 30% less than when Diet 1 was presented (Table 2).

**Figure 1:**
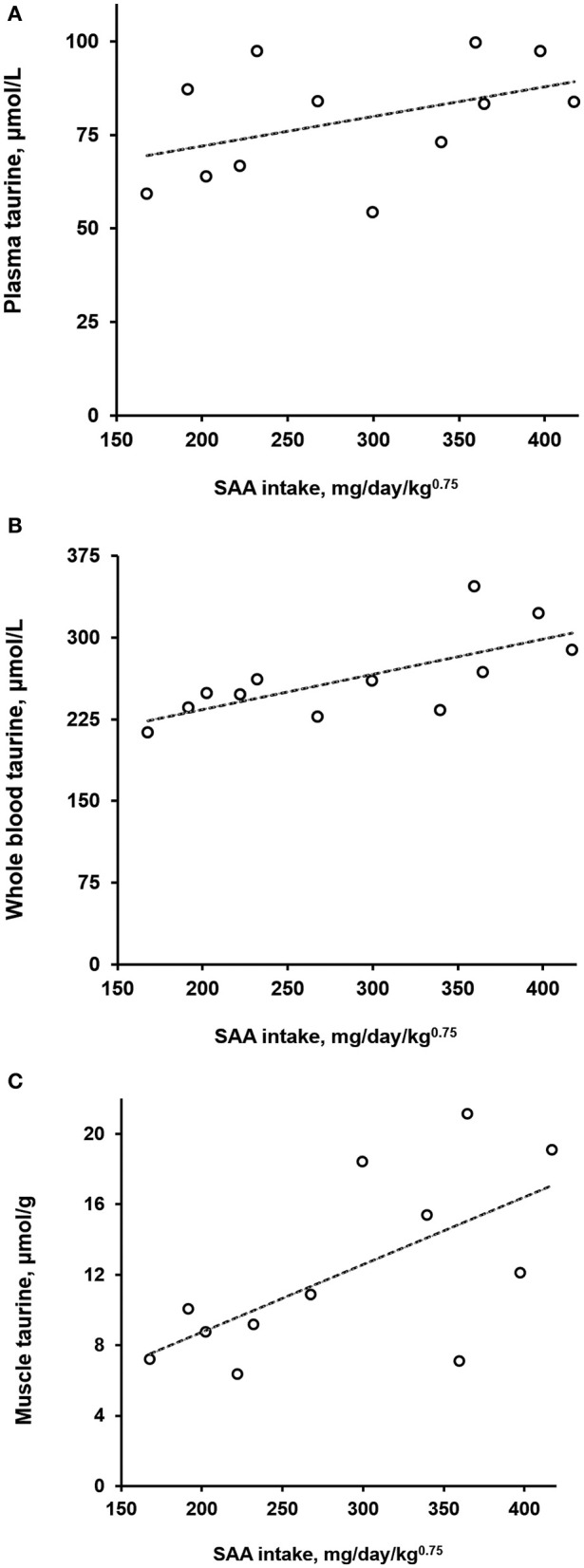
Taurine concentrations in plasma **(A)**, whole blood **(B)**, and semimembranosus muscle **(C)** of dogs as a function of daily sulfur amino acid (SAA) intake normalized by metabolic body weight. Open circles represent observations on 12 dogs given for 6 weeks a diet containing 0.85% SAA (Diet 3). Dashed lines are of plots of least-squares derived linear functions of the taurine observations on plasma, whole blood, and muscle against the normalized SAA intakes.

Normalized daily SAA intakes among the dogs varied the most during week 6 of Diet 3 presentation. Because of this, coinciding taurine concentrations in plasma, whole blood, and semimembranosus muscle were plotted against the SAA intakes (Figure 1). Significant positive correlations with SAA intake were observed for taurine concentrations in muscle (ρ = 0.64, *P* = 0.03) and whole blood (ρ = 0.73, *P* < 0.01) but not plasma (ρ = 0.39, *P* = 0.21). Linear functions derived from the observations indicated that over the range of observed SAA intakes (168–417 mg/kg^0.75^/day), whole blood taurine concentrations increased by a mean of 36% (taurine μmol/*L* = 0.324 × SAA mg/kg^0.75^/day + 169); whereas, semimembranosus muscle taurine concentrations increased by a much greater amount, a mean of 127% (taurine μmol/g = 0.038 × SSA mg/kg^0.75^/day + 1.1).

Across the diet periods, plasma taurine concentrations in SD did not significantly differ. In contrast, plasma taurine concentration in the LD declined as SAA content decreased in the diets (Table 2). When Diet 4 was presented, plasma taurine concentrations in LD were approximately 30% less (*P* < 0.01) than those in the SD.

Whole blood taurine concentrations significantly varied between diet periods (Table 2), with whole blood concentrations of LD trending downward as dietary SAA concentration decreased (Table 2). When Diet 4 was presented, whole blood taurine concentrations in SD were not significantly different from those observed when the adaptation diet and Diet 1 were received. Whereas, whole blood taurine concentrations of LD were decreased on Diet 4 compared to adaptation diet and Diet 1 by means of 25 to 12%, respectively.

Semimembranosus muscle taurine significantly changed in both dog groups during Experiment 1 (Table 2). By trial end when all dogs received Diet 4, the muscle taurine concentrations were reduced from initial values by approximately one-third (*P* < 0.01). The muscle taurine concentrations in LD were consistently less than those in SD. During presentation of Diet 4, the mean muscle taurine concentration in LD was about 60% of that in SD.

Urine taurine to creatinine ratios varied but were not significantly different between the diet periods (Table 2). Urine taurine to creatinine ratios of the LD were lower (*P* = 0.02) than those of the SD across the diet periods (Table 2).

### Experiment 2

During the 12 weeks following Experiment 1 when all dogs received oral antibiotics, body weights of SD did not significantly differ between weeks 0 and 12; which were a mean of 13.5 ± 1.3 kg over the period. Body weights of LD were greater than those of SD, and they declined (*P* < 0.01) from an initial mean of 36.3 ± 1.9 kg to a final mean of 34.4 ± 2.2 kg.

During administration of antibiotics, ME and SAA intakes, when normalized on metabolic body weight, were not significantly different between LD and SD, and did not change during the antibiotic administration.

**Figure 2:**
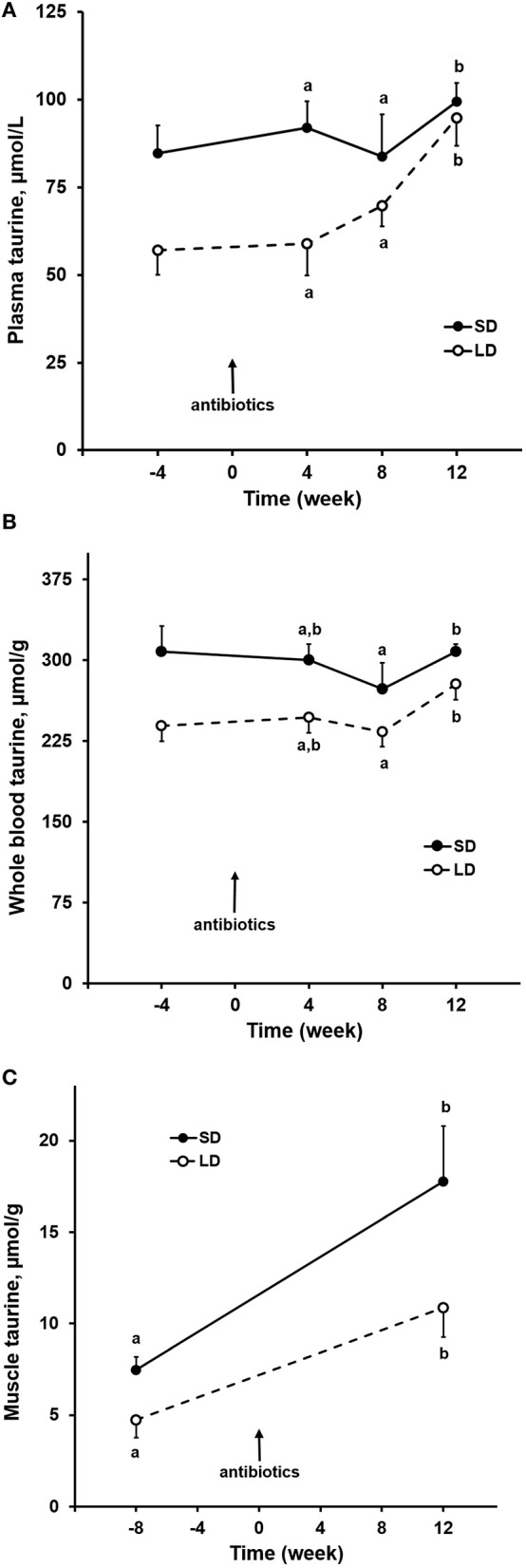
Oral antibiotics effects on taurine concentrations in plasma **(A)**, whole blood **(B)**, semimembranosus muscle **(C)** of small and large dogs. Plotted points and associated bars represent mean and SEM of observations from 6 dogs. Post-antibiotic administration, labeled points within each dog group that are without a common letter differ.

Taurine concentrations in plasma of both dog groups increased (*P* = 0.02) when the antibiotics were administered (Figure 2A). The increase was most substantial for LD, in which mean plasma taurine concentration was 80% greater after 12 weeks of antibiotic administration compared to the mean of plasma taurine concentrations 4 weeks before antibiotics administration. Taurine concentrations in whole blood during the antibiotics administration varied slightly in dogs of both groups but did not significantly increase (Figure 2B). The whole blood taurine concentrations in LD were lower (*P* < 0.01) than those in SD by a mean amount of 15%. After 12 weeks of receiving the antibiotics, taurine concentrations in semimembranosus muscle were increased (*P* < 0.01) from pre-antibiotics administration values in both dog groups by means of 130–140% (Figure 2C). Though increased, the muscle taurine concentrations remained lower (*P* = 0.02) in LD compared to SD.

When oral antibiotics were given, dry matter intake and fecal dry matter excretion were not significantly different between LD and SD when these variables were normalized by MBW (Table 3). Despite this, the concentration of bile acids in the fecal dry matter was nearly 80% higher in LD compared to SD (*P* = 0.001). The daily amount of bile acids excreted in feces when normalized by MBW was greater (*P* = 0.015) in LD compared to SD by about 50%. After 12 weeks of antibiotics, the daily fecal bile acid excretion decreased (*P* = 0.001) by approximately half for both dog groups while the apparent dry matter digestibility for both dog groups increased (*P* < 0.001) by approximately 16%.

**Table 3:** Effects of 12 weeks of oral antibiotic administration on dry matter food intake, fecal mass and digestibility, and fecal bile acid excretion in small dogs (*n* = 6) and large dogs (*n* = 6).

	**Size (*S*)**	**Time (*T*)**			***P*-value**		
**Variable^**a**^**	**Small**	**All**	**Initial**	**Week 12**	***S***	***T***	***S × T***
**Dry matter**							
Intake, g/kg^0.75^/day^*b*^	Small	40.2 ± 2.7	39.7 ± 2.8	40.7 ± 2.8	0.083	0.640	0.358
	Large	32.5 ± 2.7	31.8 ± 2.8	33.2 ± 2.8			
Fecal excretion, g/kg^0.75^/day^b^	Small	9.2 ± 0.4	11.1 ± 0.5	7.2 ± 0.5^*^	0.086	<0.001	0.911
	Large	8.2 ± 0.4	10.1 ± 0.5	6.3 ± 0.5^*^			
Digestibility, %	Small	77 ± 1	71 ± 1	82 ± 1^*^	0.258	<0.001	0.311
	Large	75 ± 1	68 ± 1	81 ± 1^*^			
**Bile acids**							
Fecal concentration, μmol/g^c^	Small	2.8 ± 0.3	3.2 ± 0.5	2.3 ± 0.5	0.001	0.109	0.781
	Large	5.0 ± 0.3^†^	5.5 ± 0.5^†^	4.5 ± 0.5			
Fecal excretion, μmol/kg^0.75^/day^b^	Small	17.6 ± 2.0	22.4 ± 2.9	12.7 ± 2.9	<0.001	0.001	0.343
	Large	26.9 ± 2.0^†^	34.7 ± 2.9	19.2 ± 2.9^*^			

a *Values are least squares means ± standard errors*.

b *Normalized by metabolic body weight*.

c *Fecal dry matter*.

* *Significantly different from initial value*.

† *Significantly different from small dog value*.

## Discussion

As a non-essential nutrient for dogs, taurine was not added to the experimental diets, hence, its concentrations in the diets were low (0.02–0.03%), only about 10–30% of recommended amounts for commercially manufactured feline diets (0.1–0.2%, 29) coming from other ingredients. In contrast, the SAA concentration (methionine + cystine) in the first experimental diet was greater than the AAFCO minimum recommendation for canine maintenance, which was raised from 0.43 to 0.65% in 2016 (38). In the last experimental diet, the SAA concentration (0.60%) was marginally low, about 90% of the current recommended minimum for commercial formulations for dogs. With respect to methionine, all experimental diets were in excess of the current AAFCO minimum recommendations (0.43–0.34 vs. 0.33%) (32). Our objective was to reduce dietary SAA content until plasma taurine concentrations of the dogs were primarily reflective of rate of taurine synthesis from SAA. When taurine is abundant, the concentration of taurine in plasma is homeostatically regulated through variation in efficiency of renal tubular reabsorption of filtered taurine (39). With scarcity of taurine, renal tubular reabsorption is at its maximum efficiency (99.5%) and a small obligatory loss of taurine occurs in urine. In this state, plasma taurine concentration should be more reflective of rate of taurine synthesis than modulation of urinary taurine loss. The mean of plasma taurine concentrations among the dogs decreased by 12% between Diets 1 and 4 suggesting that we reached our objective.

Diet 4 contained about 20% less SAA than Diet 1. In contrast, urine taurine to creatinine ratios of the dogs were not significantly different when Diets 1 and 4 were given. We suspect a significant difference was not found because of high within-individual observational variance. Taurine to creatinine ratio in a single voided urine sample may be an insensitive indicator of urinary taurine loss. The taurine to creatinine ratio is reported to be greater in dog urine with refeeding following a period of food withholding (40). Urinary amino acid excretion rates by humans are found to vary independently of creatinine excretion over 24 h (41). Therefore, differences in taurine excretion would have been better represented in taurine quantified in urine continuously collected over several 24 h.

While body weights of dogs did not significantly change, the reduction in dietary SAA concentration was associated with varied effects on concentrations of taurine in plasma, whole blood, and skeletal muscle. Across the adaptation and experimental diet periods and dog size, skeletal muscle taurine concentrations decreased by a mean of 34%, which was about twice as great as declines observed in whole blood and plasma taurine.

During presentation of the last experimental diet, Diet 4, which had the lowest SAA content, plasma taurine concentrations decreased in LD but not in SD. Over the period, plasma taurine concentrations of the LD had a mean of 62 μmol/L and ranged from 29 to 78 μmol/L with many values being low compared to the literature. Delaney and others (42) in their survey of 131 apparently healthy privately owned dogs found median plasma taurine concentration to be 78 μmol/L and the median of the lower half of observations to be 60 μmol/L. In a subsequent survey, a mean plasma taurine concentration of 81 μmol/L was reported for a greater number of subjects (*n* = 218) but limited to a single breed of large adult dogs (Newfoundlands, 43–87 kg, >2 years) (24). About 30% of these large dogs had taurine concentrations <60 μmol/L. Plasma taurine was low enough in some LD dogs to be considered taurine deficient, <40 μmol/L. The cause for the difference in plasma taurine concentrations between the LD and SD is not clear. While on the last experimental diet, SAA intakes of the dogs normalized by MBW were not significantly different between the LD and SD. For evaluation of nutrient requirements, MBW normalization is necessary as body size greatly varies across dog breeds. In dogs as in other animals, hunger is stimulated by demand for energy and is assumed to principally compel food intake. In dogs, energy intake increases with body mass (or weight) raised to exponent 0.75, this is the MBW (10). When given the same food as the only source of energy and nutrients, large dogs will eat less SAA than small dogs per kilogram of body weight while fulfilling their energy “needs”. To explain the low taurine concentrations that we observed in the LD when given Diet 4, we suggest that dietary SAA requirement of dogs may not scale in parallel with maintenance energy requirement. Supportive of this is recent research indicating the AAFCO minimum for dietary methionine maybe be too low for large dogs (43). Methionine minimum requirements for Labrador Retrievers were estimated with indicator amino acid oxidation methods to be 0.52 and 0.60% when dietary cystine concentration was greater than that of the methionine (43, 44). In our research, true digestibility of the SAA could also have differed between the dog groups. Nery and others (45) found greater amounts of protein fermentation products in feces of large compared to small dogs. To account for the finding, these investigators postulated greater passage of undigested protein to microbes of the lower gastrointestinal tract in large compared to small dogs. They postulated alternatively that body-size related differences in colonic volume, transit times, and fermentative activity could account for their findings.

During presentation of the last experimental diet, whole blood taurine concentrations did not significantly change, and the concentrations were not significantly different between the dog groups. As taurine is much less concentrated in plasma than in blood cells, particularly leukocytes and platelets (46, 47), taurine might be better conserved in blood cells of the dogs than in plasma. Observations on small cohorts of dogs diagnosed with taurine-deficiency DCM are consistent with these findings. Whole blood taurine concentrations relative to healthy population mean values are low in dogs with taurine-deficiency DCM (19, 20). Different mechanisms for regulating taurine concentration between blood cells and plasma may underlie the variation in response to taurine depletion caused by SAA restriction. Taurine concentration in blood cells is regulated by taurine uptake from extracellular fluid through expression of a high affinity taurine transporter, which is identified as TauT in several species (48). In the absence of taurine in the diet, taurine concentration in plasma is regulated by two mechanisms—by changing efficiency of reabsorption of taurine in renal tubular filtrate (39) and by varying hepatic enzymatic activities of cysteine catabolism for production of taurine (49). In the latter case, taurine is a by-product of cysteine regulation more than a target of homeostatic regulation. Dietary methionine and cystine are both substrates of taurine synthesis. Among dietary factors reportedly impacting utilization of methionine and cystine for taurine production are the sum and ratio of intakes of the amino acids relative to the quantity and quality of dietary protein (50). These observations are consistent with our finding the lowest taurine concentrations in plasma, whole blood, and muscle when dietary protein, methionine, and cystine were lowest.

The cause for greater depletion of taurine in biopsied skeletal muscle than in plasma and whole blood is not determined in this study. While dogs received the last experimental diet, a remarkable decline in taurine concentration occurred in the skeletal muscle. Mean taurine concentration in the muscle decreased by approximately 50% in the LD and 37% in the SD. The extent of these declines was surprising, especially if they were reflective of taurine depletion of the entire muscle mass, which contains the largest body pool of taurine (51). Taurine in skeletal muscle, like in blood cells, derives mostly from extracellular uptake of taurine by activity of the TauT transporter, and not from local synthesis by SAA catabolism (49, 52). The degree of reduction in muscle taurine that we observed in LD may have been functionally consequential. Abnormal morphology, atrophy, and necrosis occur in skeletal muscles of transgenic mice, in which taurine uptake is impaired by a deletion mutation of TauT (53). Skeletal muscle taurine concentrations in those mice are very low, <1.0 μmol/g, much less than the lowest muscle taurine concentrations that we observed. However, less extreme depletion of taurine might have a deleterious consequence. Investigators have produced 50 to 60% reductions in the taurine concentrations of the skeletal muscle of healthy strains of rats and mice by adding to drinking water a competitive inhibitor of taurine uptake, guanidinoethane sulfonate (54, 55). This manipulation does cause measurable dysfunctions in excitation–contraction coupling and contractile properties of skeletal muscle. It is relevant to report that this model resulted in a similar degree of taurine depletion (~60%) in cardiac muscle, a level sufficient to cause myocardial dysfunction (56). Given this, assessments of heart function and myocardial taurine concentration in retrospect would have been valuable additional outcome measures in our research, especially when taurine depletion became evident in biopsied skeletal muscle but not in plasma and whole blood. Serial determinations of taurine in biopsied myocardium would have been difficult to initially justify as it is invasive, technically demanding, and disposed to severe complications, such as, arrhythmogenesis and cardiac tamponade (57).

Our findings indicate that low taurine concentration in circulating blood may be a late indicator of taurine deficiency in dogs when the deficiency is caused by SAA restriction. This condition contrasts with taurine deficiency in cats. When cats receive diets without taurine, plasma taurine concentrations decline very rapidly and whole blood taurine concentrations decrease by half in about 5 weeks. Depletion of taurine in the muscle of the cats is much slower, decreasing by half in about 11 weeks (30). Rates of taurine loss by tissues may differ depending on the cause of taurine deficiency. In the case of low SAA intake, the taurine content of muscle may decrease even if plasma appears replete with taurine. Reduction in cysteine available to muscle may decrease intracellular synthesis of glutathione and ratio of reduced to oxidized glutathione (58). Deficiency of glutathione may damage and in turn reduce mitochondrial mass as glutathione will quench reactive oxygen species largely produced by mitochondria (59). If dietary restriction of SAA were sufficient to lessen the amount of reduced glutathione in muscle, this might diminish muscle mitochondrial mass and with it mitochondrial matrix taurine. Taurine is concentrated in mitochondria where it is suggested to buffer hydrogen ions produced by oxidative phosphorylation (60). Oxidative muscle fibers, which have a high mitochondrial density, like skeletal muscle slow-twitch fibers and myocardium, contain many times more taurine than glycolytic muscle fibers (61–63). Although the semimembranosus muscle of dogs contains a mixture of fiber types (64), skeletal muscle of dogs is unique among species in that all glycolytic fiber types are moderately to highly oxidative (65). We suggest that low dietary SAA may have reduced mitochondrial mass and thereby account for a greater decline in taurine in skeletal muscle than in plasma and whole blood. Irrespective of cause, it is noteworthy that circulating taurine concentrations may not reflect overall taurine status of dogs when dietary SAA or bioavailability is reduced. In a recent case series report, Adin and colleagues (28) ruled-out taurine deficiency as a cause of diet-associated DCM in dogs because whole blood taurine concentrations were within limits of a laboratory reference interval, 200–350 μmol/L. The whole blood taurine concentrations that we observed in the LD were within this reference range when taurine concentrations in muscle biopsies declined by half. This suggests that caution should be taken in relying on whole blood taurine alone for the diagnosis of taurine-deficiency DCM in dogs.

Low sensitivity of plasma and whole blood taurine concentrations to dietary SAA intake was also apparent with introduction of Diet 3, when SAA intake by the SD increased by a mean of about 25%. Though not intended, SAA content of Diet 3 was equivalent to that of the commercial diet, 8.5 g/kg, with the highest dietary cystine among diets fed. After presenting the diet for 6 weeks, taurine concentrations in plasma and whole blood did not significantly change, whereas taurine concentrations in the biopsied muscle increased above concentrations when SAA intake was lower at 7.4 g/kg.

Oral administration of antibiotics while feeding the last experimental diet which contained 6.0 g SAA/kg diet effectively restored taurine concentrations in plasma and muscle biopsies to concentrations observed when the first experimental diet was given, which contained 7.4 g SAA/kg diet. A similar effect of antibiotics on plasma taurine was reported when taurine-depleting diets were given to colony cats (8, 9, 66). This mechanism might also explain our observations in dogs. Taurine balance and ^14^C radiotracer kinetic studies in cats indicate that much taurine is lost to enteric microbial catabolism (14). The means by which microbes affect loss of taurine is speculative but could involve bile acids. Bile acids found in cats and dogs are conjugated almost exclusively with taurine (12, 13). Chronic oral administration of bile acid sequestrants (colestipol, cholestyramine) causes taurine deficiency in cats (67) and in dogs (68). Radiotracer studies quantifying irreversible loss of taurocholic acid in cats given taurine-depleting diets have indicated that substantive taurine losses occur in enterohepatic circulation of bile acids (14). To our knowledge similar studies have not been conducted in dogs.

Taurine balance studies in ileal-cannulated cats indicate that as much as 50% of taurine passing to the large intestine is free and not conjugated to bile acids (69). Even in cats given diet without taurine, a substantial proportion of taurine in ileal chyme is unbound (36%). Cause for this loss of free taurine is unknown but it could be an impairment of absorption mediated by microbial activity. Studies on rat and human intestinal epithelial cells show that two different amino acid transporters shuttle taurine across intestinal luminal plasma membranes. One transporter is the same as that expressed in skeletal muscle and other tissues (TauT). It has a high affinity but low capacity for taurine uptake (48). The other transporter has a low affinity but high-capacity for conveying neutral and charged amino acids. This transporter, identified as proton coupled amino acid transporter 1, is suggested to mediate bulk uptake of taurine during meals (70). Studies of this transporter have shown that indoles (71) and short-chain fatty acids (72), which are among products of microbial fermentation of dietary protein, inhibit or compete with its transport activity. We suggest that antibiotics administration to dogs in Experiment 2 may have suppressed production of microbial metabolites interfering with bulk absorption of intestinal luminal taurine, where the taurine was either ingested, freed by microbial cholyltaurine hydrolase activity, or released in turnover of intestinal epithelium, where taurine concentration is notably high (73). Such an effect might improve taurine status by diminishing taurine loss beyond that expected from bile acid loss. Lending support to microbial metabolites occurring in intestinal chyme are reports of millimolar quantities of short-chain fatty acids in the distal small intestine of sacrificed (74) and ileal-cannulated dogs following meals of nutritionally adequate diets (75, 76).

Irrespective of mechanism, the improved taurine status that we observed in response to oral antibiotics would indicate that intestinal taurine loss of dogs substantively depends on enteric microbial activity. Supportive of this conclusion would be quantification of microflora abundance before and during antibiotic administration. However, sampling from the small intestine where bile acids are actively recycled would have been unjustifiably invasive. Because taurine is synthesized from SAA andin turn utilized for many vital functions, microbial-mediated, intestinal loss of taurine also would affect the dietary SAA requirement of dogs. Aside from intestinal microbial activity, several dietary factors are reported to increase bile acid loss. Factors relevant to increasing dietary taurine requirement in cats are heat processing (6), sources and amount of dietary fiber (7), and protein source, quantity, and quality (8, 9). Pezzali et al. have recently reviewed dietary factors that might increase bile acid loss in several species (77).

Across all experimental diet periods, taurine concentrations in the biopsied skeletal muscle of the LD were significantly lower than corresponding concentrations in the SD. The evidently lower taurine status of LD compared to SD appears to be reflected in urine taurine excretion. Across all diets, urine taurine to creatinine ratio of LD was only about one third that of the SD, but the difference was not significant. Taurine concentrations in plasma and whole blood were also lower in LD compared to SD but the differences did not reach significance until diet with the lowest SAA content was fed, and then a significant difference was found only for plasma. Cause for the differences between dog sizes remains to be determined. Taurine-deficiency DCM and diet-associated DCM are more frequently diagnosed in large than small-breed dogs (20, 26, 27). It may be that intestinal loss of taurine from inefficient recycling of bile acids in the small intestine increases with dog size. Our finding that fecal bile acid concentrations and excretions in the LD were greater than in the SD are consistent with this hypothesis and agree with findings of others. For our SD, fecal bile acid concentrations (2.8 ± 0.3 μmol/g) are similar to those reported by Pezzali et al. for Beagles (2.7 ± 0.8 μmol/g) (77). Donadelli et al. report total fecal bile acid concentrations for large dogs, Labrador Retrievers (78). With conversion of their units from μg/mg to μmol/g (assuming bile acid molecular weight of 409 g/mol), mean fecal bile acid concentration was 4.25 μmol/g when a “grain-based, control” diet was fed. We observed mean fecal bile acid concentration among the LD to be 5.0 μmol/g. Large compared to small dogs may in general have a more limited small intestinal capacity for absorption of bile acids and other substances, including nutrients. Feces of large compared to small dogs contain greater concentrations of moisture, electrolytes, and microbial fermentation products (45). Of possible explanations for this observation, Weber et al. (79) postulated a greater flow of undigested chyme entering the large intestine of large dogs than small dogs, as well as body size differences in microbiota populations and colonic residence time for fermentation.

Limitations of our research were that a small number of dogs were studied and that dietary groups, although balanced for dog size, were not contemporaneously evaluated. A crossover design could have been used. However, this approach would have been prohibitively costly and of long duration with potential carryover effects of varying initial taurine status and prior antibiotic administration. Our findings on rate and extent of depletion of taurine may or may not have reflected global changes in skeletal muscle or other tissues, including cardiac muscle. Nonetheless, withdrawal of dietary taurine from cats or ingestion of taurine antagonist by rats cause depletion of taurine in all evaluated body tissues, albeit at different rates (80, 81). Total fecal bile acid excretion values that we report may be low because feces of the dogs, although stored at −20 °C prior to analyses, were collected once daily rather than immediately following passage. Some bacterial degradation of bile acid structures may have occurred before feces were collected. The relevance of our findings may be limited to taurine depletion caused by intake of a diet marginal in bioavailable SAA with no added taurine. Many manufacturers of commercial dog foods now supplement their products with taurine in response to reports of diet-associated DCM in dogs (82). It remains to be determined whether dietary taurine supplementation may correct in all tissues, depletion resulting from inadequate SAA intake. An evaluation is needed to ascertain the long-term healthfulness of taurine supplementation to compensate for inadequate SAA intake and the role of the microbiota.

In conclusion, we observed that restriction of dietary SAA concentration to 3.4% methionine and 2.6% cystine depleted taurine in skeletal muscle at a greater rate than in plasma and whole blood. The rate and extent of taurine depletion was greater in large than small dogs. We additionally observed that oral administration of antibiotics corrected taurine depletion without affecting excretion of bile acids in feces. We conclude that intestinal taurine loss in dogs may depend on enteric microbial activity to the extent that taurine status and dietary SAA requirement are substantively impacted. We further conclude that declining taurine concentrations in plasma and whole blood may not be sensitive indicators of a developing taurine deficiency in dogs. Caution is advised in using circulating taurine concentration for ruling-out taurine deficiency as contributory or causal of DCM. Future investigations should evaluate effects of SAA restriction on skeletal and cardiac muscle function when taurine status is controlled and made adequate by dietary taurine supplementation.

## Data Availability Statement

The raw data supporting the conclusions of this article will be made available by the authors, without undue reservation.

## Ethics Statement

Protocols for use of the dogs (8,840 and 9,550) were approved by the Animal Use and Care Committee at the University of California, Davis and complied with the NRC guidelines for research animals.

## Author Contributions

CT conducted the experimental trials. CT and RB compiled and analyzed the data, authored the manuscript, and prepared the figures and tables. RB performed the statistical analysis and had primary responsibility for the final content. All authors contributed to the conception and design of the research, interpreted the results, and reviewed the final manuscript.

## Funding

This study and CT were supported by a grant from Royal Canin, SAS, France in 2001. At this date, Royal Canin was not part of Mars, Inc. The experimental diets were designed and produced with the help of Royal Canin, SAS. Additional funding was obtained from the Center for Companion Animal Health, University of California, Davis, Davis, CA and the Morris Animal Foundation, Englewood, CO.

## Conflict of Interest

This study received funding from Royal Canin, SAS, France. The funder had the following involvement with the study: production and analysis of diets used and intellectual contributions to design of experimental trials, interpretation of results, and writing of the report. The authors declare that the research was conducted in the absence of any commercial or financial relationships that could be construed as a potential conflict of interest.

## Publisher's Note

All claims expressed in this article are solely those of the authors and do not necessarily represent those of their affiliated organizations, or those of the publisher, the editors and the reviewers. Any product that may be evaluated in this article, or claim that may be made by its manufacturer, is not guaranteed or endorsed by the publisher.

## Abbreviations

AAFCO: Association of American Feed Control Officials
BCS: body condition score
DCM: dilated cardiomyopathy
LD: large dogs
MBW: metabolic body weight
ME: metabolizable energy
SAA: sulfur amino acid
SD: small dogs
TauT: taurine transporter.
